# Association of Depression/Anxiety Symptoms with Neck Pain: A Systematic Review and Meta-Analysis of Literature in China

**DOI:** 10.1155/2018/3259431

**Published:** 2018-09-25

**Authors:** Fushui Liu, Ting Fang, Fanyuan Zhou, Meimei Zhao, Mei Chen, Jianyu You, Yuli Jin, Jinmei Xie, Zhongyong Liu

**Affiliations:** ^1^School of Moxibustion, Jiangxi University of Traditional Chinese Medicine, Nanchang, China; ^2^College of Clinical Medicine, Jiangxi University of Traditional Chinese Medicine, Nanchang, China

## Abstract

**Background:**

Due to its high morbidity and prevalence, the potential relationships of depression/anxiety symptoms in neck pain (NP) are not well demonstrated.

**Objectives:**

This study aimed to conduct a comprehensive estimation of controlled trials of psychological problems and to test hypotheses concerning whether NP was statistically relative to anxiety/depression symptoms.

**Methods:**

Chinese literature databases such as the China National Knowledge Infrastructure (CNKI), VIP Information (VIP), Chinese Biomedicine (CBM), and Wanfang Data (WANFANG) were scientifically searched for reports published until February 5, 2018. Controlled trials incorporating NP patients with anxiety/depression versus healthy people were contained. Two researchers screened each article and extracted data, respectively, and blinded to the findings of each other. Meta-analysis was conducted by the Cochrane Collaboration's RevMan 5.3 and Stata 14.0 (Stata Corp LP, USA) software.

**Results:**

We identified 13 eligible studies involving 2339 patients and 3290 healthy people. Compared with healthy control participants, the findings indicated that depression/anxiety symptoms were more common or severe in NP patients (respectively, SMD = 0.89; 95% CI = (0.58, 1.20); *P* < 0.01 and SMD = 0.92; 95% CI = (0.65, 1.20); and *P* < 0.01), results from the pooled data demonstrated no statistical significance between depression/anxiety symptoms and gender in NP patients (resp., SMD = 0.16; 95% CI = (−0.18, 0.51); *P*=0.35 and SMD = −0.08; 95% CI = (−0.42, 0.27); and *P*=0.67), and the combined data of the incidence of depression or anxiety symptoms revealed significant difference between NP patients and healthy persons (resp., RR = 4.81; 95% CI = (3.30, 7.01); *P* < 0.01 and RR = 3.29; 95% CI = (2.16, 5.00); and *P* < 0.01). In addition, we did not find articles that met the inclusion criteria, which compared NP patients with other physical illnesses in terms of anxiety/depression symptoms.

**Conclusions:**

This meta-analysis suggests that anxiety/depression symptoms are associated with high morbidity in NP patients. We consider these reports support the viewpoint that nonspecific mechanisms mediate mental disturbances in NP. This study may have clinical value for NP, offering an underlying target for the prevention and treatment of anxiety/depression.

## 1. Introduction

Neck pain (NP) has become a common public health problem all over the world, with a high rate of disability, presents a negative impact on the health and quality of life in people, and exerts great pressure on individuals, families, health systems, and social economy [1–3]. Reportedly, NP was ranked the fourth leading cause of disability next to ischemic heart disease, cerebrovascular disease, and lower respiratory infection, with an annual prevalence rate exceeding 30% [4, 5]. In 2005, over a third of a billion had experienced neck pain more than 3 months of duration [4, 5]. Preliminary statistics showed that the incidence of NP in China ranged between 3.8% and 17.6%, and it affected nearly 15% of the global population [6, 7]. NP has obvious financial problems and the annual economic losses caused by NP are as high as $5 billion in America, while it can reach $800 million just for the treatment cost per year in China [6]. The prevention and treatment of NP has attracted the wide attention of people from all walks of life and has important practical significance for the study of NP.

The clinical symptoms of NP are complicated, which mainly include neck, shoulder and back pain, stiffness and weakness of upper limbs, numbness of fingers, dizziness, nausea, vomiting, and even blurred vision [8]. According to its clinical symptoms, it is divided into three syndromes, namely, cervical radiculopathy, cervical myelopathy, and axial neck pain [9]. By contrast, it contains seven types in China, including neck type, nerve root type, vertebral artery type, sympathetic type, spinal cord type, mixed type, and other type (mainly include esophageal compression type) [10]. The clinical diagnosis of NP is established on the basis of the identification of positive symptoms via diagnostic criteria and the exclusion of organic illness, for instance, unexplained dizziness and numbness [10]. Treatment for NP mainly depends on lifestyle intervention, medications, and physical therapies [11].

Research has found that mental health disorders are always connected with physical illness [12, 13] and is also more common in developing than developed countries [14]. What is particularly exciting here is the abundant description of depression/anxiety symptoms in NP. However, scholars differ in opinions on the nature of their relationships [15, 16]. In short, at present, it exists two opposing schools of thought: one considers that NP may be tied closely to depression/anxiety through some pathogenesis and the other considers the associations between NP and mental disorders as nonspecific or chance events.

Despite plentiful descriptions have been reported about mental disturbs in NP patients, no systematic evaluation of the relationship between NP and depression/anxiety symptoms has been found. This article is to systematically review prospective cohort or case-control studies and investigate the association between NP and anxiety/depression symptoms. We completed the quality assessment of included studies to analyze any inconsistencies in data. The research questions are (1) whether depression/anxiety symptoms are more common or serious in NP patients than in healthy people or patients with other medical diseases? and (2) does depression/anxiety symptoms differ from gender in NP patients?

## 2. Methods

### 2.1. Search Strategy

According to Cochrane Reviews' Handbook, we developed a study protocol for this systematic review and meta-analysis [17]. Electronic searches were systematically performed in CNKI, VIP, CBM, and WANFANG databases up to February 5, 2018, by two authors (Ting Fang and Mei Chen). Medical subject headings (MeSH) terms relevant to neck pain, anxiety, and depression was utilized in the search, and the same terms in Chinese databases. Then, we browsed the abstracts and full-text articles, respectively, and picked the eligible studies in line with the inclusion criteria. Additionally, we also manually searched the relevant lists of all eligible studies to identify further potential studies and contacted reference authors for additional data if necessary.

### 2.2. Criteria for Selecting Articles

Inclusion criteria include (1) prospective cohort or case-control studies that concerned anxiety/depression symptoms measured at baseline and their relationship with neck pain; (2) population-based studies that have compared a group of persons with neck pain with another group of either healthy persons or persons with other medical illness; (3) studies that evaluated depression and anxiety symptoms via validated psychometric testings or a structured clinical interview; (4) people diagnosed with neck pain utilizing a certain diagnostic criterion and patients' gender, age, the source of the case, and duration of illness were not limited; and (5) studies must have provided reasonable data for estimating effect size and confidence intervals.

Exclusion criteria include duplicate studies, animal experiments, no clear diagnostic criteria for NP, the measures of anxiety/depression symptoms were not standardized, studies that included treatment measures; and studies that only included NP patients as a subgroup of a large sample and not compared, respectively, from the other participants.

### 2.3. Data Extraction

Data were independently extracted from the eligible studies by two authors (Ting Fang and Mei Chen) and cross checked. Any discrepancies were discussed among Fushui Liu, Meimei Zhao, and Fanyuan Zhou. The key information was collected systematically using a predefined Excel template. It mainly included first author, year of publication, case source, baseline characteristics for participants (age, sex, and number of participants), diagnostic criteria of NP, assessment standards of depression and anxiety symptoms, and outcome assessments. Where possible, we contacted the first author for clarifying the ambiguous information that provided in some studies.

### 2.4. Quality Assessment

For detecting bias of included studies, the methodological quality was assessed according to a validated rating scale [18], which was used in psychiatric case-control studies. For this meta-analysis, we regulated this scale and researched selection bias of cases (seven items), selection of bias of controls (six items), and information bias (one item). The quality assessments were completed independently by two reviewers (Ting Fang and Mei Chen). Disagreements would be settled by discussing and analyzing between reviewers.

### 2.5. Data Analysis

We used RevMan 5.3 statistical software (the Nordic Cochrane Centre, Copenhagen; the Cochrane Collaboration, 2014) for meta-analysis. We defined *P* ≤ 0.05 as statistically significant between studies. We calculated combined risk ratio (RR) with 95% confidence intervals (CI) for the categorical data; as continuous variables, we estimated combined standard mean difference (SMD) with 95% CI. The studies' heterogeneity was evaluated by the chi-square test and Higgins *I*^2^ test, and when *I*^2^ ≤ 50% and *P* ≥ 0.10, the fixed effect model was used or else the random effect model was applied.

### 2.6. Ethical Statement

All analyses were based on previously published studies, and so ethical approval was superfluous.

## 3. Results

### 3.1. Search Results

**Figure 1:**
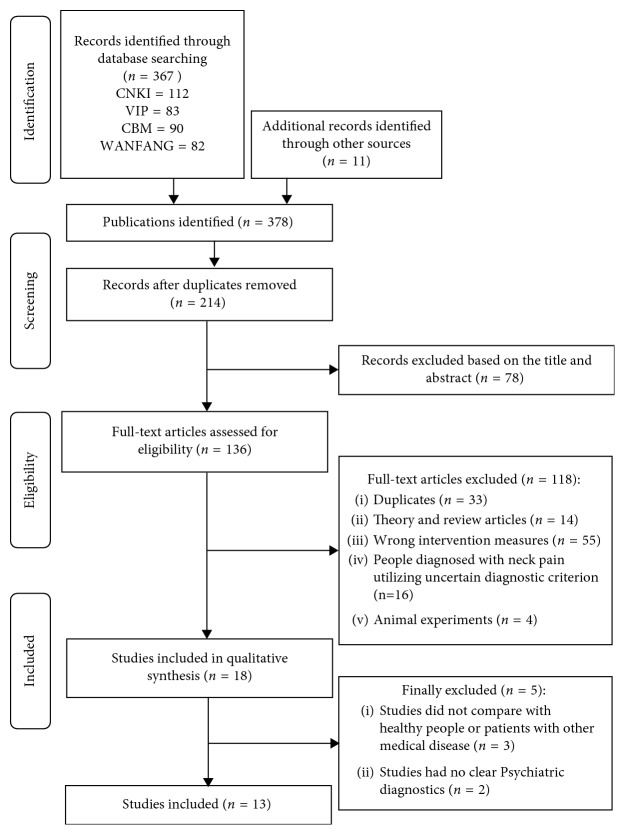
Flow diagram outlining the selection of studies for the review.

Totally, 378 potential literature citations were initially obtained through database examinations. We removed 164 duplicates with EndNote software. 78 articles were ruled out through scanning the title and abstracts. And 118 articles were eliminated according to the exclusion and inclusion criteria. In the rest 18 articles, 3 of them did not compare with healthy people or patients with other medical disease, 2 studies failed to have assessment standards of depression and anxiety symptoms, and 13 eligible articles [7, 19–30] were included finally. The whole selection process is shown in Figure 1.

### 3.2. Basic Characteristics of Eligible Studies

The eligible studies were published between 2002 and 2017 in China, and 2339 patients and 3290 healthy controls were included. In these studies, regarding the source of cases, three studies [7, 22, 24] were multiple-center controlled trials, two studies [19, 26] were ambiguous, and the remaining studies were single-center controlled trials. Also, they were all completed in outpatients and inpatients, except one [29] which was completed in college students. As for the source of cases in control groups, seven groups [7, 19, 20, 25–28] were from domestic normal population, and the others were healthy people.

NP was diagnosed in 4 studies [22, 25, 29, 30] using the diagnostic criteria acknowledged in China, and the others used self-rating criteria which were validated and reliable. As for the diagnostic criteria of depression and anxiety symptoms, all the studies reported validated scale. Seven studies [7, 19–21, 23, 25, 26] utilized the symptom check list-90 (SCL-90), and 1 study [24] used the symptom check list-290 (SCL-290) to assess depression and anxiety symptoms levels; among them, one study [19] also used the state-trait anxiety inventory (STAI). One study [30] utilized generalized anxiety disorder-7 (GAD-7) and patient health questionnaire-9 (PHQ-9) as the evaluation standard, 4 studies [22, 24, 27, 28] utilized self-rating depression scale (SDS), 2 studies [24, 27] used self-rating depression scale (SAS), and the criteria in the last one [29] were unclear.  Table 1 shows the key characteristics of the included studies: study design, sample sizes, design, mean ages, diagnosis criteria for NP, and scales utilized for assessments of the levels of anxiety and depression.

### 3.3. Quality Assessment

Table 2 reports the methodological quality of the included studies. The clinical setting used for recruitment was frequently reported eligible information, so were the inclusion/exclusion criteria for cases. As for controls, one item concerning the using of students or employees of the research institution was always rated as “no,” indicating good methodological practice. Few studies provided sufficient information about the using of advertising for recruitment. The rest questionnaire items for cases and controls were poorly described. Approximately half studies could not offer enough information on whether the investigators were “masked” or not.

### 3.4. Depression/Anxiety Symptoms Levels in NP Patients: Continuous

**Figure 2:**
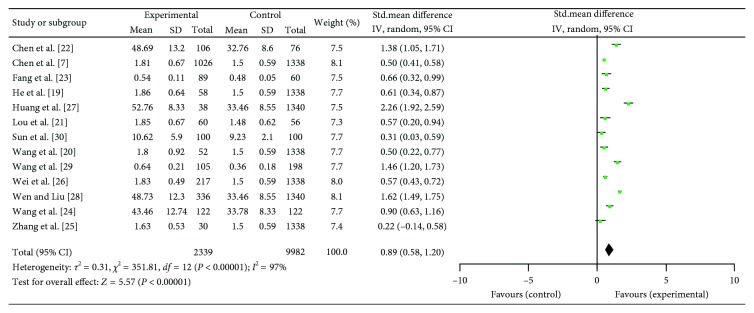
Meta-analysis of 13 studies about depression in NP.

**Figure 3:**
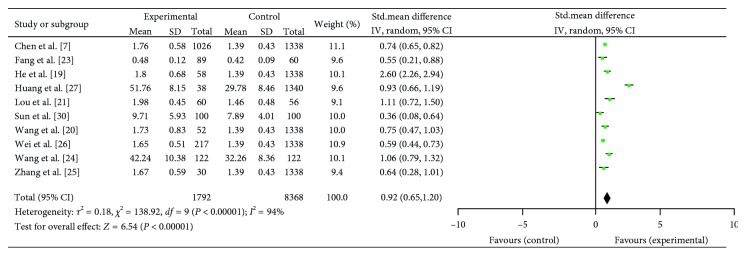
Meta-analysis of 10 studies about anxiety in NP patients.

Depression and anxiety scores were, respectively, achieved in 13 and 10 studies that included SCL score and other depression rating scores. Data extracted from both reported significantly heterogeneity in the consistency of study results (respectively, *I*^2^ = 97%, *P* < 0.01, and *I*^2^ = 94%, *P* < 0.01), and the random effects model was applied. Overall, the depression and anxiety symptoms' scores were distinctly higher in NP patients compared to healthy control groups (resp., SMD = 0.89; 95% CI = (0.58, 1.20); *P* < 0.01 and SMD = 0.92; 95% CI = (0.65, 1.20); *P* < 0.01) (Figures 2 and 3).

### 3.5. Relationship between Anxiety/Depression Symptoms and Gender in NP Patients: Continuous

**Figure 4:**
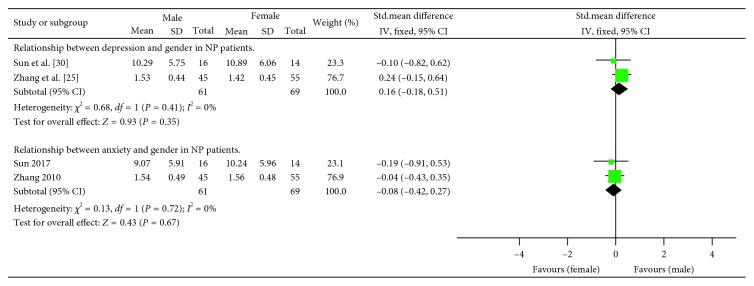
Relationship between depression/anxiety and gender in NP patients.

Only 2 studies [25, 30] reported the relationship between depression/anxiety scores and gender in NP patients. No heterogeneity exist in this analysis, and the fixed effects model was used. The overall results demonstrated no significant difference (resp., SMD = 0.16; 95% CI = (−0.18, 0.51); *P* = 0.35 and SMD = −0.08; 95% CI = (−0.42, 0.27); *P* = 0.67) (Figure 4).

### 3.6. The Incidence of Depression/Anxiety Symptoms in NP Patients: Categorical

**Figure 5:**
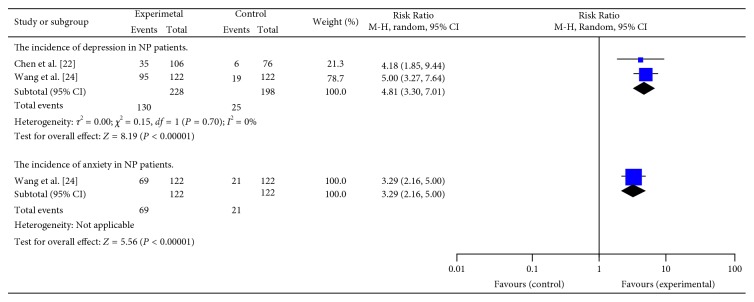
The incidence of depression/anxiety in NP patients.

5 studies [22, 24, 27, 28, 30] provided categorical data for depression and anxiety, but only 2 of them [22, 24] presented categorical data in detail. The combined data revealed significant difference between NP patients and healthy persons, indicated that depression and anxiety were closely linked to NP patients (resp., RR = 4.81; 95% CI = (3.30, 7.01); *P* < 0.01 and RR = 3.29; 95% CI = (2.16, 5.00); *P* < 0.01) (Figure 5).

### 3.7. Sensitivity Analysis for Continuous Data

We completed the same meta-analysis for depression symptoms level in NP patients excluding 6 studies [22, 24, 27–30] whose psychiatric diagnostics were distinctly different from the remaining studies. The level of depression symptoms of NP patients was still higher than healthy control groups (SMD = 0.52; 95% CI = (0.45, 0.58); *P* < 0.01). More importantly, the *I*^2^ showed no heterogeneity between studies (I2 = 0%, *P* = 0.59). We also conducted the same meta-analysis for anxiety level in NP patients excluding 3 studies [24, 27, 30], as the psychiatric diagnostics were different from the remaining studies. The *I*^2^ drops from 95 to 44%, *P* = 0.10, and the level of anxiety remained practically stable (SMD = 0.73; 95% CI = (0.62, 0.84); *P* < 0.01). Totally, our different sensitivity analyses presented similar findings and indicated that the different psychiatric diagnostic criteria resulted high risk for the results.

## 4. Discussion

In this study, we identified 13 eligible studies involving 2339 patients and 3290 healthy people that intended to evaluate possible links between NP and mental disorders. According to our findings, compared to healthy control groups, the depression and anxiety symptoms scores were distinctly higher in NP patients. However, there was no significant difference between depression/anxiety symptoms and gender in NP patients. And we failed to obtain case reports to confirm whether NP patients differ from other chronic diseases in terms of anxiety and depression symptoms. But we found articles from PubMed which testified significant difference between NP patients and other chronic physical illnesses in terms of anxiety and depression symptoms [31, 32], and this conclusion deserved further exploration in future. Also, this study suggested that the morbidity of depression and anxiety symptoms was higher in NP patients. Wen and Liu [28] reported that the morbidity of depression symptoms in 336 cases of NP patients was 36.31%, Huang et al. [27] found that incidence of anxiety and depression symptoms in 38 NP patients was 60.53% and 92.11%, respectively, while it was 77% and 84%, respectively, in Sun's survey of 100 NP patients [30].

Neck pain is a common and frequently occurring disease with complicated clinical etiology, which is mainly related to people's bad living habits, such as long-term head bending posture. It is easy to relapse and lasts for a long time [33]. Long-term chronic pain has a profound impact on patients' mental health, and they are prone to negative emotions such as anxiety and depression, which seriously affect patients' quality of life. Reportedly, the longer the chronic pain lasts, the more severe it becomes and the more anxious or depressive it becomes [34]. Meanwhile, anxiety and depression can also promote psychological responses to chronic pain [34].

Some authors investigated the effects of depression and anxiety symptoms in NP patients and found NP and poor quality of life were two factors leading to depression and anxiety [35]. Instead, Galbusera and Gorter reported depression and anxiety symptoms were two major factors affecting the quality of life in patients with musculoskeletal pain [36, 37]. Relatively, few were currently known about causal mechanisms for depression and anxiety symptoms in NP patients. Different mechanisms perhaps could explain the relationship between pain and anxiety from different aspects of biology, psychology, and sociology [38]. Functional imaging studies showed the affective processing area of brain in patients with anxiety and depression symptoms that changed from the insula topology to the prefrontal area of weight-bearing pain management [35]. Besides, it was also found patients with depression and anxiety symptoms had dysfunction of autonomic nervous function and inflammation and activity hyperactivity of the hypothalamic-pituitary-adrenal (HPA) axis [38–41]. Interestingly, when neurotransmitters such as norepinephrine (NE) and 5-hydroxytryptamine (5-HT) decrease, the inhibitory mechanism of pain can be impeded and the development of affective disorders can be promoted [42]. Additionally, studies found an increase in systemic inflammatory markers in the blood of patients with pain disorders and affective disturbances, which suggests that there is the same basic pathogenetic pathway between the two diseases [43–45]. Similarly, consideration from the view of psychological sociology, pain, and mental disorders are also closely related. Many studies point out that psychological stress and potential obstacles caused by pain can possibly cause immunological changes that eventually results in depression and anxiety [40, 45–47]. In addition, a study had shown that both depression and pain might be risk factors for each other [48].

Anyhow, we should notice potential restrictions of this article. It included first that although we assessed the methodological quality of the included studies via a rating scale [18] for case-control studies, we cannot declare that the rating scale is valid even if the score presented high credibility. Second, few studies provided categorical data, despite 5 studies [22, 24, 27, 28, 30] had involved it, and none of them provided complete data except 2 [22, 24]. The incomplete data may not draw definite conclusion. Furthermore, there were no uniform inclusion and exclusion criteria, which may be a source of heterogeneity.

Despite these limitations, there are still many advantages in our study. In order to improve the accountability of the systematic procedure of this systematic review, we had taken several steps. Two reviewers (Ting Fang and Mei Chen) conducted the electronic searches and picked the suitable studies after browsing the abstracts and full-text articles, respectively, according to the inclusion criteria. Data extraction was also completed, respectively, by two authors. And the methodological quality of the included studies was still evaluated by two authors, and the results were counted.

## 5. Conclusions

Overall, NP is characterized as a kind of chronic degenerative disease, which has a series of serious influences on body and psychology and severely reduces the quality of life of patients. Therefore, attention should be cautiously attached to the psychological problems in the treatment of NP patients. And clinicians should give the necessary psychological counseling and psychological treatment, which may help alleviate the mental pain of patients and relieve their physical pain at the same time. Moreover, clinicians who treat pain syndromes should not only improve the patients' correct understanding of disease and treatment but also strengthen patients' confidence to overcome the disease.

## Figures and Tables

**Table 1:** Summary of studies included in the meta-analyses on anxiety and depression in NP.

Study	Sample sizes	Design	Group comparison	Mean age	NP diagnostics	Psychiatric diagnostics
He et al. [19]	58 vs. 1 388	Case-control	NP vs. Np	49.5	Self-rating	SCL-90/STAI
Wang et al. [20]	52 vs. 1 338	Case-control	NP vs. Np	48.25 ± 13.36	Self-rating	SCL-90
Lou et al. [21]	60 vs. 56	Case-control	NP vs. HC	54.31 ± 8.18	Self-rating	SCL-90
Chen et al. [22]	106 vs. 76	Case-control	NP vs. HC	51 ± 8 vs. 48 ± 7	Chinese acknowledged diagnostic criteria	SDS
Fang et al. [23]	89 vs. 60	Case-control	NP vs. HC	49.3 vs. 47.6	Self-rating	SCL-90
Yao et al. [24]	122 vs. 122	Case-control	NP vs. HC	58.86 ± 8.28 vs. 59.36 ± 7.04	Self-rating	SCL-290/SDS/SAS
Zhang et al. [25]	30 vs. 1 338	Case-control	NP vs. Np	52	Chinese acknowledged diagnostic criteria	SCL-90
Wei et al. [26]	217 vs. 1 338	Case-control	NP vs. Np	38.00 ± 5.67	Self-rating	SCL-90
Huang et al. [27]	38 vs. 1 340	Case-control	NP vs. Np	/	Self-rating	SDS/SAS
Wen and Liu [28]	336 vs. 1 340	Case-control	NP vs. Np	/	Self-rating	SDS
Wang et al. [29]	105 vs. 198	Prospective cohort	NP vs. HC	/	Chinese acknowledged diagnostic criteria	Self-rating
Chen et al. [7]	1 026 vs. 1 338	Case-control	NP vs. Np	/	Self-rating	SCL-90
Sun et al. [30]	100 vs. 100	Case-control	NP vs. HC	45.98 ± 8.54 vs. 45.86 ± 8.43	Chinese acknowledged diagnostic criteria	GAD-7/PHQ-9

NP, neck pain; HC, healthy controls; Np, normal population; SCL, symptom check list; STAI, state-trait anxiety inventory; SDS, self-rating depression scale; SAS, self-rating anxiety scale; GAD-7, generalized anxiety disorder-7; and PHQ-9, patient health questionnaire-9.

**Table 2:** Methodological quality of the case-control studies (*N* = 12).

Question	Answer		
	Yes	No	Unclear
	*N*	*N*	*N*
*Cases*			
Was the clinical setting used for recruitment made clear?	10	1	1
Was the denominator from which cases were recruited described?	8	1	3
Was duration of illness adequately described?	4	8	0
Was adequate information given on the total number of patients approached?	3	9	0
Was information given on participants and nonparticipants?	2	10	0
Was information given on the differences between participants and refusers?	0	12	0
Were the inclusion and exclusion criteria described well enough to be replicable?	10	2	0

*Controls*			
Did the study use controls who were students/employees of the research institution?^*∗*^	0	11	1
Were controls selected from an explicit sampling frame?	9	2	1
Did the study recruit through advertisements?^*∗*^	0	11	1
Were similar exclusion criteria applied for controls as for cases?	4	0	8
Was information given on number of controls approached?	6	6	0
Was adequate information given on differences between controls refusing and agreeing?	0	12	0
Information bias			
Were the investigators who rated the exposure masked to participants' status?	9	3	0

^*∗*^“no” is the answer indicative of good methodological practice.
